# KDM6A downregulation promotes tumor-prone cytokines expression in cancer-associated fibroblasts by activating enhancers

**DOI:** 10.1038/s41419-025-07818-3

**Published:** 2025-07-14

**Authors:** Jieying Zhang, Suoyu Xiang, Dan Liu, Xiaomeng Pei, Meng Chen, Yiheng Zhao, Yongbin Wang, Qiong Wang, Lan Kang, Zuoren Yu, Jun Mi, Wujun Xiong

**Affiliations:** 1 Hongqiao International Institute of Medicine, Tongren Hospital; Basic Medical Institute; Key Laboratory of Cell Differentiation and Apoptosis of the Chinese Ministry of Education, Shanghai Jiao Tong University School of Medicine, Shanghai, China; 2 Research Center for Translational Medicine, Shanghai East Hospital, Tongji University School of Medicine, Shanghai, China; 3 Institute of Cancer Stem Cell, Dalian Medical University, Dalian, China; 4 Institute for Regenerative Medicine, Shanghai East Hospital, Shanghai Key Laboratory of Signaling and Disease Research, School of Life Sciences and Technology, Tongji University, Shanghai, China; 5 Department of Gastroenterology, Shanghai Pudong Hospital, Fudan University Pudong Medical Center, Shanghai, China

**Keywords:** Cancer microenvironment, Cancer metabolism

## Abstract

Cancer-associated fibroblasts (CAFs) are activated fibroblasts that secrete numerous cytokines and chemokines to accelerate tumor progression. However, the mechanism underlying cytokine production by CAFs remains unclear. This study reports that CAFs isolated from colon cancer tissue, TGF-β1-induced CAFs, or HCT116 co-cultured CAFs secrete more cytokines and growth factors represented by IGF1, ELN, and SFRP2. Mechanistic investigations demonstrate that aerobic glycolysis metabolites fumarate and succinate can induce the transcription of IGF1, ELN, and SFRP2 in CAFs, while α-ketoglutarate (α-KG) can antagonize the induction effect of fumarate and succinate. Moreover, the downregulation of KDM6A in CAFs is observed compared to quiescent fibroblasts (NAFs). Additionally, integrated analysis of ATAC sequencing and RNA sequencing revealed altered chromatin structure during fibroblast activation. CUT-tag sequencing and co-IP assays demonstrate that KDM6A is bound to WDR5, facilitating its association with the COMPASS complex and the polycomb repressive complex at the expected target loci. Depletion of KDM6A disrupts the homeostasis between polycomb and COMPASS complexes, leading to an increase in the expression of IGF1, ELN, and SFRP2. However, the inhibitor GSK-J4, specific for both KDM6A and KDM6B, reduces IGF1 expression, indicating that KDM6B compensates for the demethylase function of KDM6A but cannot replace KDM6A to maintain the homeostasis of COMPASS and polycomb repressive complexes. These findings suggest a metabolism-related epigenetic mechanism for cytokine expression, where reduced KDM6A levels enhance the tumor-promoting effect of CAFs. This may provide insights into why colon cancer is more prevalent in men than in women, since KDM6A is an X-chromosome-associated gene.

## Introduction

Activated fibroblasts, also known as carcinoma-associated fibroblasts (CAF), are key components of the tumor stroma. CAFs play critical roles in tumor initiation, progression, and metastasis by secreting a range of cytokines/chemokines and ECM-degrading proteases [1–7]. The specific cytokines/chemokines produced by CAFs can vary depending on their origin and subpopulation. Noteworthy factors that have been extensively investigated include HGF, IGF1/2, PDGF, TGF-β, VEGF, and SDF1. Among these, IGF1 and HGF/Met signaling predominantly drive proliferation, migration, invasion, and angiogenesis across various tumor types. Furthermore, CAFs contribute to the resistance of cancer cells to chemotherapy and radiotherapy and promote tumor relapse through paracrine release of cytokines after radiotherapy [8–11]. For instance, IGF1 has been found to enhance resistance to anti-EGFR therapy in lung cancer [8, 12–17].

In contrast, quiescent fibroblasts (NAFs) typically express low levels of cytokines and chemokines, raising questions about the mechanisms that enable CAFs to produce these molecules at much higher levels. Recent studies suggest that CAFs undergo metabolic reprogramming, often engaging in aerobic glycolysis [18–22]. However, it remains unclear whether and how this metabolic shift directly influences the production of cytokines/chemokines.

KDM6A/B is a Fe^2+^- and α-ketoglutarate-dependent demethylase that removes specific di- and tri-methylation groups from histone H3K27, a modification associated with the polycomb repressive complex 2 (PRC2) [23–25]. Beyond its H3K27 demethylase activity, KDM6A is also involved in the modulation of gene transcription through interactions with various epigenetic complexes. It can form a complex with the H3K4 methyltransferase MLL3/4, the histone acetyltransferase p300/CBP, and the retinoic acid receptors (RARs) and retinoid X receptors (RXRs) heterodimer, thereby regulating H3K4me1/3, H3K27 acetylation levels, which control enhancer activation [26–28]. KDM6A, also known as ubiquitously transcribed X chromosome tetratricopeptide repeat protein (UTX), is the homolog of UTY on the Y chromosome. Notably, KDM6A exhibits significantly higher enzymatic activity compared to UTY, and consequently, its expression is generally higher in females than in males, correlating with the number of X chromosomes present [29–31].

Our findings indicate that the downregulation of histone demethylase KDM6A alters chromatin structure by disrupting the methylation and demethylation of H3K27 (dimethylation/trimethylation), which in turn promotes cytokine expression in colorectal CAFs. This occurs through the activation of enhancers, suggesting that KDM6A plays a structural role in regulating cytokine expression, beyond its histone demethylase activity.

## Results

### Cytokines upregulation in colorectal cancer-associated fibroblasts

**Fig. 1:**
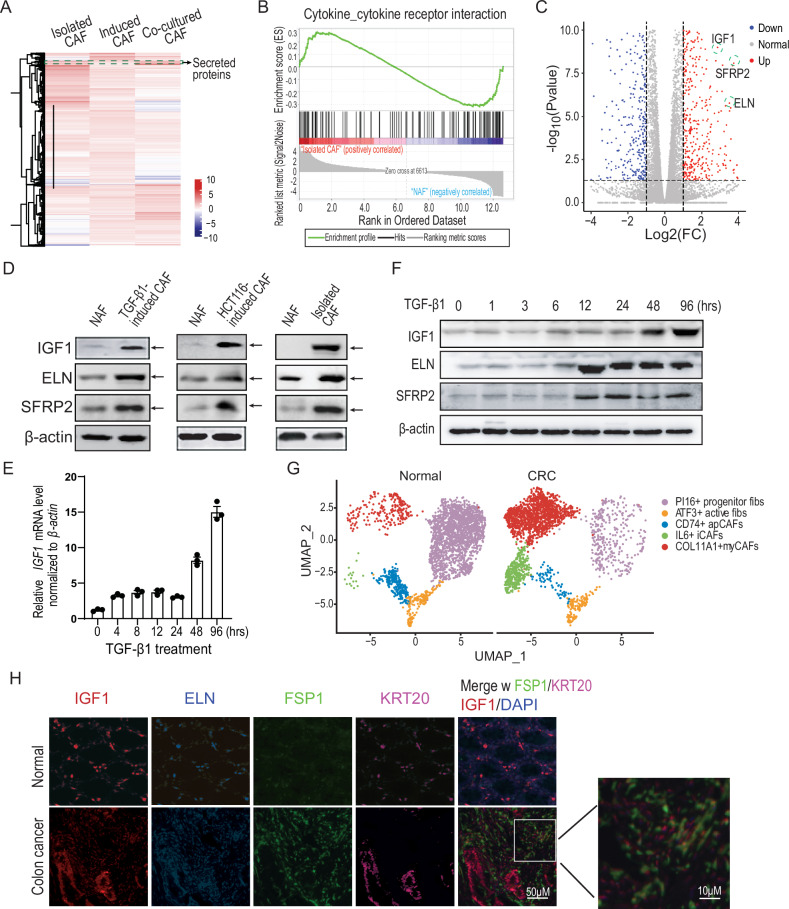
Cytokines were upregulated in colorectal cancer-associated fibroblasts. **A** RNA expression profile heatmap of inactivated fibroblasts from different sources. The isolated CAFs: CAFs isolated from clinical colorectal cancers; the induced CAFs: primary non-activated fibroblasts (NAFs) treated by TGF-β1; the co-cultured CAFs: primary non-activated fibroblasts co-cultured with HCT116 cells. Primary non-activated fibroblasts were isolated from the paratumor colon. **B** GSEA analysis of genes in the cytokine–cytokine receptor pathway between the isolated CAFs and NAFs. **C** The volcano graph of differential RNA expression between the isolated CAFs and NAFs. **D** Detection of the expression of IGF1, ELN, and SFRP2 cytokines in cancer-activated fibroblasts, and TGF-β1- and PDGF-activated fibroblasts. The concentration of TGF-β1 and PDGF was 10 ng/ml. **E** Q-PCR detection of IGF1, ELN, and SFRP2 expression in NAFs treated by TGF-β1 at the indicated time points. **F** Immunoblotting detection of IGF1, ELN, and SFRP2 expression in NAFs treated withTGF-β1 at the indicated time points. **G** Analysis of cell type subpopulations in paratumor or colorectal carcinoma by single-cell sequencing. **H** Multiple-color IHC staining of the expression of IGF1 and ELN cytokines in colon cancers. The green is FSP1 staining, a marker of CAFs, while the purple is KRT20 staining, a marker of cancer cells.

To investigate the cytokine expression profile, we conducted RNA-sequencing on colorectal cancer-associated fibroblasts (CAFs) derived from isolated CAFs, TGF-β1-induced CAFs, and HCT116 co-culture-induced CAFs. The results revealed a significant upregulation of gene expression, particularly for secreted proteins, in all three sources compared to the non-activated fibroblasts (NAFs), as shown in the heatmap (Figs. 1A and S1A). Gene set enrichment analysis (GSEA) further demonstrated the activation of cytokine–cytokine receptor interaction signaling in all three types of CAFs studied (Figs. 1B and S1B). Notably, compared to quiescent fibroblasts, expression of IGF1, SFRP2, and ELN was significantly upregulated in CAFs (Fig. 1C), a finding that was corroborated by immunoblotting in all three CAF types (Fig. 1D).

Further analysis of the expression of these genes during CAF transformation showed that both mRNA and protein levels did not significantly increase until 48 h post-TGF-β1 treatment, at which point CAF transformation began (Fig. 1E and F). Single-cell sequencing analysis revealed that the predominant fibroblast subtype in colorectal carcinoma was myofibroblast (Fig. 1G). Additionally, immunofluorescence staining demonstrated that in colon cancer, both ELN and IGF1 were primarily expressed in CAFs, with some of IGF1 also detected in cancer cells (Fig. 1H). This suggests that cytokine expression is a fundamental characteristic of CAF, not solely dependent on TGF-β1 treatment.

### Decreased effective α-KG enhances cytokine expression in CAFs

**Fig. 2:**
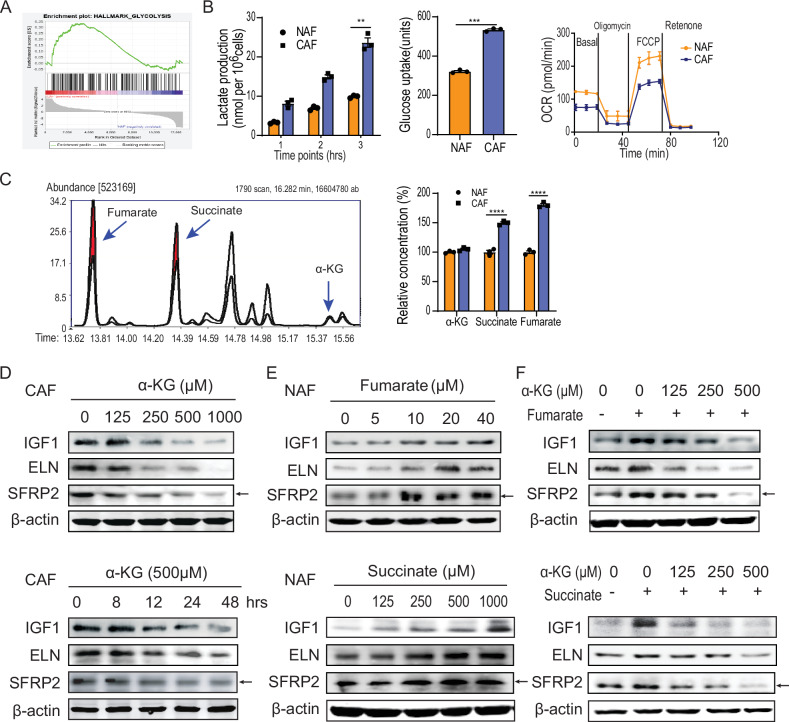
The decrease in effective α-KG upregulated cytokines expression in CAFs. **A** The GSEA analysis of the genes involved in glycolysis between the isolated CAFs and NAFs. **B** Detection of lactate production, glucose uptake, and oxygen consumption in the CAFs and NAFs. The lactate concentration was measured by an ELISA kit; the glucose uptake was determined by mass-spectrometry following incubation with ^13^C-labeled glucose; the oxygen consumption was analyzed by the Seahorse. **C** Analysis of TCA (the citric acid cycle) intermedia metabolites in CAFs and NAFs by the LC–MS/MS. The red area represented the disparity in the content of fumarate, succinate, and α-KG between the isolated CAFs and NAFs. **D** Immunoblotting detection of IGF1 expression in CAFs treated with the α-KG at the indicated concentrations (0, 125, 250, 500, 1000 µM) and the indicated time points. **E** Immunoblotting detection of IGF1 expression in NAFs treated with fumarate or succinate at the indicated concentration. The concentration of fumarate ranges from 0 to 40 µM, while the concentration of succinate ranges from 0 to 1000 µM. **F** Immunoblotting detection of IGF1 expression in NAFs treated with the fumarate or the succinate and increasing concentration of α-KG.

Recent studies have indicated that CAFs undergo a metabolic shift towards aerobic glycolysis, similar to tumor cells [18, 32]. This metabolic reprogramming in CAFs may contribute to the enhanced expression of cytokines and chemokines. To confirm the presence of glycolytic activity in CAFs, we conducted a GSEA analysis on glucose metabolism. The results demonstrated an upregulation of glycolysis signaling in CAFs derived from three different sources (Figs. 2A and S2A). This was further supported by increased lactate production and extracellular acidification, enhanced glucose uptake, and reduced oxygen consumption in CAFs (Figs. 2B and S2B).

To investigate whether glycolysis drives cytokine expression in CAFs, we conducted a quantitative analysis of glucose catabolites using LC–MS/MS. Our data revealed a significant increase in the concentration of fumarate and succinate in colorectal CAFs compared to control fibroblasts. In contrast, the concentration of α-ketoglutarate (α-KG) exhibited only a modest change (Fig. 2C). Notably, the ratio of effective α-KG regulates gene expression by modulating dioxygenases activity, consistent with previous studies [24, 33–36].

We then examined the impact of individual fumarate, succinate, and α-KG on cytokine expression in fibroblasts. As shown in Fig. 2D, we observed a time- and dose-dependent decrease in the expression of IGF1, ELN, and SFRP2 in response to α-KG in CAFs. In contrast, fumarate and succinate increased the expression of these cytokines in a dose-dependent manner in NAFs (Fig. 2E). However, when fumarate and/or succinate were combined with increasing concentrations of α-KG, cytokine expression gradually decreased (Figs. 2F and S2C). These results suggest that glycolysis enhances cytokine expression in CAFs by reducing the effective α-KG.

### KDM6A depletion promoted cytokine expression in CAFs

**Fig. 3:**
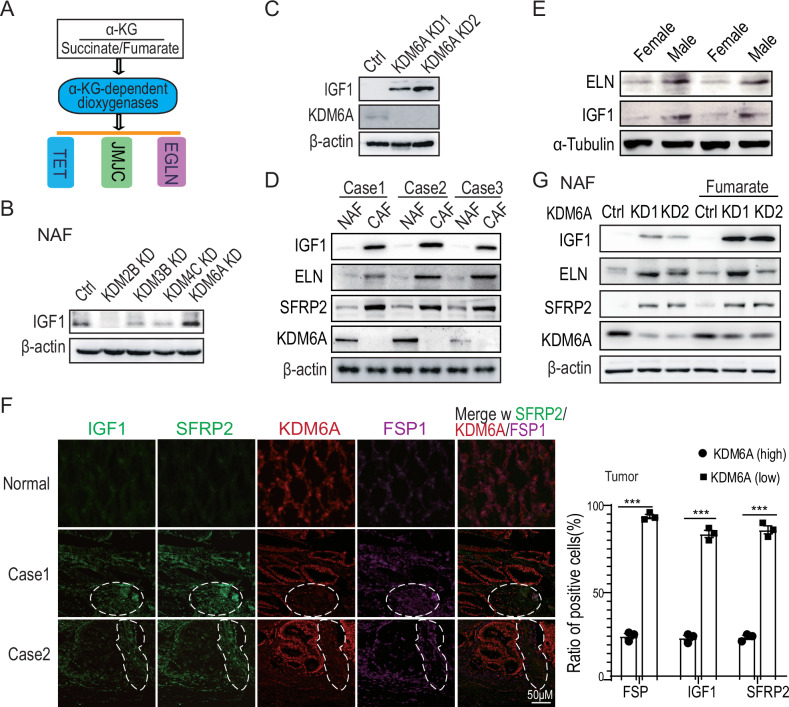
KDM6A depletion promoted cytokines expression in CAFs. **A** A schematic illustration of the mechanism of effective α-ketoglutarate regulation on dioxygenases, such as TET, JMJC, and EGLN subfamilies. **B** Immunoblotting verification of IGF1 expression in NAFs depleted of candidate demethylases via shRNAs. **C** Immunoblotting detection of IGF1 expression in NAFs depleted of KDM6A using two specific shRNAs. **D** Immunoblotting detection of IGF1, ELN, SFRP2, and KDM6A expression in CAFs isolated from clinical colon cancers, in comparison to the paired paratumoral NAFs. **E** Immunoblotting detection of ELN and IGF1 expression in either male or female colorectal carcinomas. **F** The in situ immunofluorescence staining in clinical colon cancers. The FSP1 is a marker of CAFs; the Red represents KDM6A and the green represents IGF1. **G** Immunoblotting detection of IGF1, ELN, and SFRP2 expression in NAFs depleted of KDM6A with or without α-KG treatment. The concentration of α-KG was 1 mM.

It is widely acknowledged that α-KG regulates the activities of a group of dioxygenases [18, 37–40] (Fig. 3A). These dioxygenases utilize α-KG as a cofactor to catalyze hydroxylation reactions on a variety of substrates, including proteins, alkylated DNA and RNA, lipids, antibiotics, and 5-methylcytosine in genomic DNA [41, 42]. To identify the particular dioxygenase(s) responsible for regulating cytokine expression in CAFs, we individually silenced 24 dioxygenases using targeted shRNAs. Our quantitative PCR results (Figs. S3A and S3B) indicate that the knockdown of KDM2B, KDM3B, KDM4C, or KDM6A led to an increase in IGF1 expression compared to the control shRNA. However, only the depletion of KDM6A significantly elevated IGF1 protein levels in fibroblasts, as confirmed by immunoblotting. This finding was further validated using multiple antisense shRNAs targeting KDM6A (Figs. 3B and S3C).

Immunoblotting analysis of clinical colorectal cancer samples revealed that KDM6A expression was markedly reduced in CAFs compared to non-activated fibroblasts (NAFs) from the adjacent non-cancerous tissue. In contrast, the levels of SFRP2, ELN, and IGF1 levels were higher in CAFs, as demonstrated in Fig. 3D. Additionally, protein levels of ELN and IGF1 were found to be more abundant in CAFs from male patients than from female patients (Fig. 3E). In situ immunofluorescence staining displayed an inverse correlation between KDM6A expression and the levels of IGF1, ELN, and SFRP2 in colorectal cancers, as illustrated in Fig. 3F. Furthermore, treatment with fumarate increased the expression of IGF1, ELN, and SFRP2 in KDM6A-competent NAFs, but had no significant effects in KDM6A-deficient NAFs. This is shown in Fig. 3G, indicating that KDM6A plays a critical role in the α-KG-mediated regulation of cytokine expression.

### KDM6A depletion enhances cytokines’ expression by activating enhancers

**Fig. 4:**
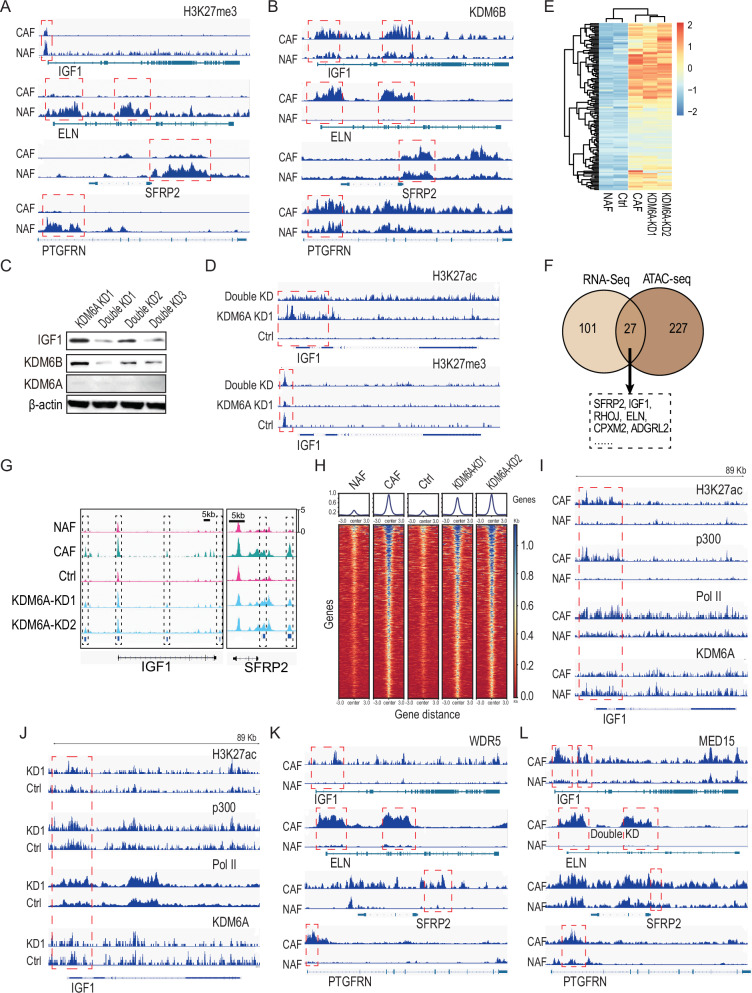
KDM6A depletion enhanced cytokines’ expression by activating enhancers. **A** Analysis of H3K27me3 enrichment at the loci of *IGF1*, *ELN, SFRP2*, and *PTGRN* genes. **B** Analysis of KDM6B enrichment at the loci of *IGF1*, *ELN, SFRP2*, and *PTGRN* genes. **C** Immunoblotting detection of IGF1 expression in fibroblasts depleted of KDM6A and KDM6B. **D** Examination of H3K27 acetylation and tri-methylation enrichment at the locus of *IGF1* gene. **E** The heatmap of RNA expression profile in various fibroblasts. **F** The overlap of upregulated genes from RNA-sequencing analysis and the enriched genes from ATAC-sequencing analysis. **G** The peaks at the upregulated genes analyzed by ATAC-sequencing. **H** Evaluation of KDM6A enrichment at the whole genomic range in CAFs, NAFs, KDM6A-depleted fibroblasts, and control fibroblasts. **I** Assessment of the H3K27 acetylation, p300, RNA polymerase II, and KDM6A enrichment at the locus of *IGF1* gene in CAFs and NAFs. **J** Assessment of the H3K27 acetylation, p300, RNA polymerase II, and KDM6A enrichment at the locus of *IGF1* gene in KDM6A-depleted fibroblasts and control fibroblasts. **K** Analysis of the WDR5 enrichment at the loci of *IGF1*, *ELN, SFRP2*, and *PTGRN* genes by Cut-Tag technology. **L** Analysis of the Med15 enrichment at the loci of *IGF1*, *ELN, SFRP2*, and *PTGRN* genes by Cut-Tag technology.

Although KDM6A is deficient or lost in CAF cells, a reduction in H3K27 trimethylation was observed at the promoter region of the four target genes in CAFs (Fig. 4A). This raised the question of why KDM6A downregulation did not lead to an increase in H3K27 methylation. We speculated that another H3K27 demethylase, KDM6B, which did not show altered protein levels in CAFs (Fig. S4A), might compensate for KDM6A by removing H3K27 di- and tri-methylation. Our data supported this hypothesis, demonstrating that KDM6B was enriched at the promoters of IGF1, ELN, SFRP2, and PTGFRN and that this binding was further enhanced upon KDM6A depletion (Figs. 4B and S4B). Moreover, double depletion of KDM6A and KDM6B resulted in a complete loss of IGF1 expression, unlike KDM6A depletion alone (Fig. 4C). This was accompanied by reduced H3K27 acetylation peaks and increased H3K27 trimethylation peaks, further supporting our hypothesis that KDM6A depletion upregulates cytokine expression through mechanisms independent of its demethylase activity (Fig. 4D).

To investigate how KDM6A affects the expression of IGF1, we integrated ATAC-seq and RNA-seq to analyze the chromatin accessibility in NAFs, CAFs, and KDM6A-depleted NAFs. RNA-seq revealed that the expression profiles of CAFs and KDM6A-depleted NAF were similar but significantly different from NAFs (Fig. 4E). ATAC-seq analysis showed increased chromatin accessibility at the loci of 27 upregulated genes, including SFRP2, IGF1, ELN, CPXM2, and PTGFRN, in both CAFs and KDM6A-depleted NAFs (Fig. 4F). These peaks were enhanced at the loci of these upregulated genes in CAFs and KDM6A-depleted NAFs compared to NAFs (Figs. 4G and S4C), suggesting that KDM6A depletion alters chromatin structure to facilitate gene activation.

To determine whether KDM6A depletion activates enhancers and enhances cytokine expression, we conducted a CUT-Tag (Cleavage Under Targets and Tagmentation) analysis using specific antibodies against H3K27Ac, KDM6A, RNA polymerase II, and p300. The results revealed reduced KDM6A binding at the loci of target genes in both CAFs and KDM6A-depleted NAFs (Fig. 4H). In contrast, peaks of H3K27Ac, p300, and RNA polymerase II were enriched across the broad loci of IGF1 and ELN genes in both CAFs (Figs. 4I and S4C) and KDM6A-depleted NAFs (Figs. 4J and S4D), indicating that enhancer activation is involved in regulating cytokine expression in these cells.

We further explored whether KDM6A depletion promotes cytokine expression by recruiting the COMPASS complex to the upregulated gene loci. Protein co-immunoprecipitation assays showed that KDM6A was associated with WDR5, Med15, and p300 (Fig. S4E). Additionally, CUT-Tag analysis revealed that WDR5 and Med15 were enriched at the loci of IGF1, ELN, SFRP2, and PTGRGN in CAFs (Fig. 4K and L). Furthermore, H3K4 trimethylation was increased at the promoter regions of these genes in CAFs (Fig. S4F). These findings suggest that KDM6A recruits the COMPASS complex to the promoters of upregulated genes, supporting the previous observation that KDM6A loss activates super-enhancers [43].

### KDM6 depletion promoted tumor formation of colon cancer in vitro and in vivo

**Fig. 5:**
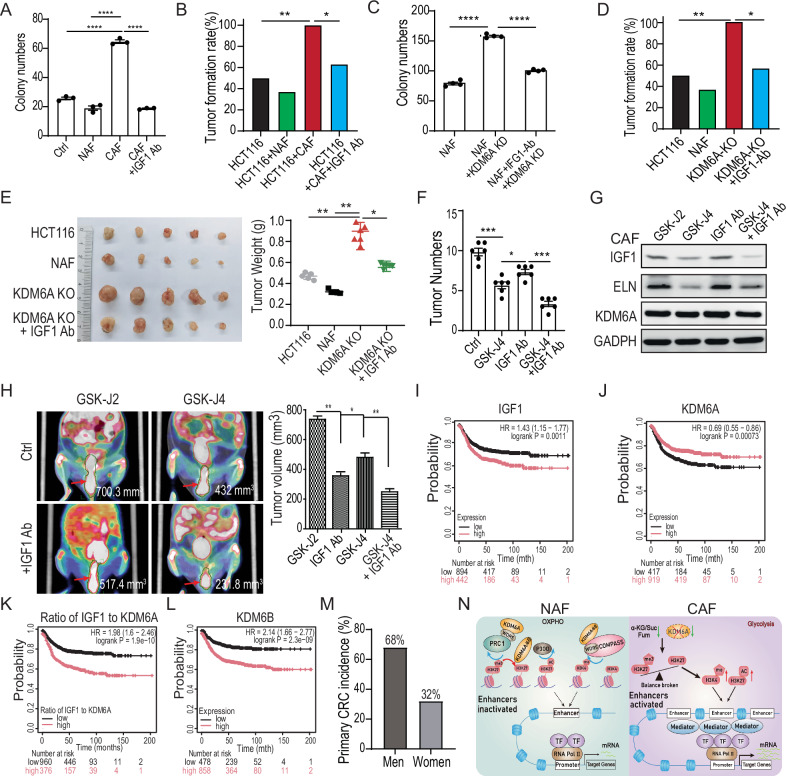
KDM6 depletion promoted tumor formation of colon cancer in vitro and in vivo. **A** Determination of the impact of IGF1 on HCT116 cell growth by the colony formation assay. HCT116 cells were co-cultured with fibroblasts-conditioned media treated with or without the IGF1 neutronization antibody. The concentration of IGF1 neutralization antibody was 1 μg/ml. The 5 × 10^2^ of HCT116 cells were initially seeded. **B** Assessment of the effect of IGF1 on tumor formation in a xenograft mouse model. The 5 × 10^5^ of HCT116 cells and 1 × 10^6^ NAFs or CAFs were co-injected. The mice were sacrificed 4 weeks after injection. The dose of the IGF1 neutralization antibody was 10 mg/kg. There were nine mice in each group. **C** Detection of the influence of KDM6A depletion on HCT116 cell growth by the colony formation assay. The HCT116 cells were co-cultured with NAF- or CAF-conditioned media. The NAFs were depleted of KDM6A by Crisp-Cas9 technology. **D** Assessment of the effect of KDM6A on the tumor formation in a xenograft mouse model. The 5 × 10^5^ of HCT116 cells and 1 × 10^6^ NAFs or NAF depleted of KDM6A were co-injected. The observation ended 2 weeks after the injection. There were twelve mice in each group. **E** Determination of the influence of KDM6A depletion on tumor growth in subcutaneous xenografts. The 5 × 10^5^ of HCT116 cells and 1 × 10^6^ NAFs or NAF depleted of KDM6A were co-injected. The mice were sacrificed 4 weeks after injection. There were seven mice in each group. **F** Evaluation of the impact of KDM6 inhibitor on tumor formation in an AOM/DSS-induced mouse tumor model. The doses of GSK-J2 and GSk-J4 were 10 mg/kg. **G** Analysis of the expression of IGF1 and ELN in xenografts after the designated treatments. **H** The representative ^18^F PET-CT images of tumors induced by AOM/DSS in mice. The treatments were similar to those mentioned above. **I**–**L** The Kaplan–Meier plotter analysis between the expression level of IGF1, KDM6A, and KDM6B, and the ratio of IGF1 to KDM6A expression level and the overall survival of colorectal cancer patients. The total number of patients was 1336. The IGF1, KDM6A, and KDM6B analysis cutoff values were 175, 207, and 117, respectively. **M** The incidence of primary colorectal cancer in males and females. **N** Our working model. The **P < 0.01; the #*P* > 0.05. The concentration of the IGF1 neutronization antibody was 10 mg/kg, and the concentration of GSK-J2 and GSK-J4 was 20 and 10 μM, respectively.

The IGF1 pathway interacts with genetic and environmental factors known to contribute to cancer development [40, 41]. As shown in Figs. 5A and S5A, colony formation assays demonstrated that colon cancer cells co-cultured with CAFs formed more colonies compared to those co-cultured with NAFs. Neutralizing IGF1 with an antibody significantly reduced the number of colonies formed by colon cancer cells. Additionally, both IGF1 and CAF-conditioned media enhanced the resistance of HCT116 cells to mitomycin C (Fig. S5B and S5C). In a xenograft mouse model, CAFs promoted tumor formation in all nine mice, while NAF led to tumor formation in only six out of nine mice. Treatment with an IGF1 neutralization antibody decreased tumor formation, with tumors appearing in only seven out of nine mice. Injection of colon cancer cells alone resulted in similar tumor numbers as when co-injected with NAFs (Fig. 5B).

Next, we assessed the role of KDM6A depletion in fibroblasts on colon cancer formation. Our findings revealed that colon cancer cells co-cultured with KDM6A-depleted fibroblasts formed more colonies than those co-cultured with NAFs. However, the use of an IGF1 neutralization antibody reduced colony formation (Figs. 5C and S5D). Furthermore, KDM6A-depleted fibroblasts significantly increased tumor formation in xenografted HCT116 cells from 50% (6/12) to 100% (12/12). However, the IGF1 neutralization antibody reversed this effect, reducing tumor formation from 100% to 58.8% (7/12) (Fig. 5D). In subcutaneous xenograft mice, KDM6A depletion significantly promoted tumor growth, and treatment with the IGF1 neutralization antibody reduced this tumor-promotion effect (Fig. 5E).

In a colon cancer model induced by AOM (azoxymethane)/DSS (dextran sulfate sodium), the KDM6A/B-specific inhibitor GSK-J4 markedly reduced the number of tumors (13 per mouse) compared to the inactive GSK.J2 (22 per mouse). Furthermore, the IGF1 neutralization antibody significantly reduced tumor number in both the antibody-alone group (10 per mouse) and the GSK-J4 combination group (3.5 per mouse) (Figs. 5F and S5E). Immunoblotting results presented in Fig. 5G indicated that the KDM6 inhibitor GSK-J4 suppressed the expression of IGF1 and ELN in AOM/DSS-induced colon cancer but had minimal effect on KDM6A protein levels. Representative PET-CT images revealed that the control group (GSK-J2) displayed higher ^18^F-FDG uptake, while both the IGF1 antibody and GSK-J4 treatment reduced the ^18^F-FDG uptake (Fig. 5H), consistent with the observed results.

Additionally, Kaplan–Meier survival analysis demonstrated that patients with high IGF1 expression had significantly shorter overall survival compared to those with lower IGF1 expression (*p* = 0.0011). In contrast, high KDM6A expression was associated with a favorable survival (*p* = 0.00073) (Fig. 5I and J). The elevated ratio of IGF1 to KDM6A expression further predicted a more pronounced disparity in patient survival (Fig. 5K). Conversely, high expression of KDM6B predicted a worse outcome in terms of patient survival (Fig. 5L). As anticipated, the incidence of primary colorectal cancer was significantly higher in males than in females (68% vs. 32%) (Fig. 5M). These findings emphasize the potential of the KDM6 inhibitor as an effective treatment approach for colorectal cancer.

## Discussion

Cancer-associated fibroblasts (CAFs) play a crucial role in tumor progression by secreting a wide range of cytokines and chemokines. Unlike tumor cells, CAFs maintain genomic integrity, and their robust cytokines/chemokines expression is likely regulated epigenetically during the transformation of fibroblasts into CAFs.

Previous research has indicated that CAFs undergo metabolic reprogramming and increasingly rely on glycolysis [18, 22]. Additionally, CAFs exhibit reduced levels of effective α-KG, a metabolic modulator that may influence gene expression by regulating dioxygenase activity [18]. In our study, we found that the expression of cytokines/chemokines in CAFs is controlled by effective α-KG. This metabolic regulation is mediated through the activation of super-enhancers, which are inhibited by the histone demethylase KDM6A/B. Inhibition of KDM6A/B led to increased cytokine/chemokine expression by enhancing the activation of these super-enhancers.

KDM6A is a critical protein in the regulation of gene expression. It removes methyl groups from repressive histone markers, specifically H3K27me2 and H3K27me3. These histone modifications, also regulated by the polycomb repressive complex 2 (PRC2), mark genes involved in essential cellular processes such as development, differentiation, and proliferation [44–46]. Additionally, KDM6A plays a vital role in the COMPASS complex by binding to enhancer loci, which drives the robust expression of target genes. For instance, it upregulates HOX genes during the differentiation of teratocarcinoma and embryonic stem cells [47], suggesting that KDM6A functions both through its demethylase activity and structural role in chromatin regulation [48–52]. Moreover, KDM6A is frequently mutated in various human cancer types, with most mutations being loss-of-function mutations [53]. However, how KDM6A regulates gene expression independently of its demethylase activity remains an open question.

KDM6A associates with both the COMPASS complex and the polycomb repressive complex via binding to WDR5. In contrast, the closely related demethylase KDM6B only associates with the COMPASS complex [54–56]. When KDM6A is deficient, this may disrupt the balance between the polycomb and COMPASS complexes, particularly during fibroblast activation. In this context, KDM6B compensates by removing methyl groups from H3K27, but unlike KDM6A, KDM6B cannot recruit the polycomb repressive complex to the target gene. This imbalance leads to a reprogramming of the chromatin structure, altering the methylation status of H3K27. Consequently, the COMPASS complex activates enhancers or super-enhancers, leading to the substantial upregulation of target gene expression.

Interestingly, our findings may also provide insight into the higher incidence and mortality rates of colorectal cancer in males compared to females (Fig. 5M). The lower expression of KDM6A in males could contribute to this disparity, as the altered chromatin structure and enhanced cytokine expression may promote tumorigenesis.

In conclusion, our work demonstrates that the downregulation of KDM6A alters chromatin structure by disrupting the balance of methylation and demethylation of H3K27, leading to the upregulation of tumor-promoting cytokines. This regulation relies on the structural role of KDM6A in the activation of enhancers. These findings suggest a molecular mechanism that may explain the higher incidence and mortality of colorectal cancer in males compared to females (Fig. 5N).

## Methods and materials

### Reagents and antibodies

Fetal bovine serum (FBS) was purchased from Gibco (USA), GSK-J2 was purchased from Med Chem Express (USA), and GSK-J1 and GSK-J4 were purchased from TargetMol (USA). SYBR Green master mix was from eBioscience (USA). The α-Ketoglutarate Colorimetric/Fluorometric Assay Kit was purchased from BioVision Technologies (USA), and the SimpleChIP® Enzymatic Chromatin IP Kit (Magnetic Beads) was purchased from Cell Signaling Technology (USA). The detailed antibody information was referred to the Supplementary Table 1: Antibody list.

### Cell culture and lentivirus infection

Primary human fibroblasts (non-activated fibroblasts, NAFs) and cancer-associated fibroblasts (CAFs) were isolated from normal colon tissue from the surgical incisal margin or colon cancer tissue, respectively, following previously described methods [57].

HEK293T and HCT116 cells were cultured in DMEM (Gibco, USA) supplemented with 10% FBS (Gibco, USA) at 37 °C in a 5% CO_2_ incubator. HEK293T and HCT116 cells were used in this study and obtained from our laboratory, and identified by STR and tested for mycoplasma contamination. The control shRNA plasmid and two KDM6A shRNAs were transfected into HEK293T cells using pSPAX2 and pMD2G for lentivirus preparation. The shRNA-mediated knockdown was performed according to previously described protocols [33].

### Quantitative real-time PCR (qRT-PCR)

Total cellular RNA was extracted using the RNeasy mini kit (QIAGEN, USA). According to the manufacturer’s instructions, 1 µg of RNA was synthesized into cDNA using the PrimeScript RT Reagent kit (TaKaRa, Japan) for mRNA quantification. qRT-PCR was performed using the 7500 Sequence Detector (Applied Biosystems by Life Technologies, USA) with Power SYBR Green PCR Master Mix (EBioscience, USA). The β-actin gene was used as an endogenous control for normalization.

### Western blotting

The cells were harvested and washed with PBS. Then, RIPA buffer (Thermo Fisher, USA) and protease inhibitor cocktail (Roche, Switzerland) were added, and the cells were incubated on ice for 30 min. Protein extracts were collected by centrifugation. The BCA assay kit determined the protein concentration (Ding Guo Biotechnology, China). Next, the proteins were analyzed by SDS–polyacrylamide gel electrophoresis and transferred to a nitrocellulose or polyvinylidene fluoride membrane. The blots were blocked in 5% non-fat milk and incubated with antibodies overnight at 4 °C. The secondary antibody was purchased from TransGen Biotech (China).

### Immunostaining

Tumor samples were collected at East Hospital, Tongji University, with written informed consent obtained from all participants. Immunofluorescence staining was performed on cells, paraffin-embedded tissue sections, or OCT medium-embedded tissue sections. For paraffin-embedded sections, they were deparaffinized with xylene and rehydrated through successive washes of ethanol and water (starting from 100% ethanol and gradually decreasing to 95% ethanol). Cells and frozen sections were fixed in 4% paraformaldehyde on ice for 20 min. After permeabilization with 0.4% Triton-X100 for 10 min, the tissues were blocked with 10% goat serum in PBS for 30 min. Cells or tissue sections were then incubated with primary antibodies overnight at 4 °C. The opalTM 4-color fIHC kit (PerkinElmer, USA) was used according to the manufacturer’s instructions. For immunohistochemistry, after incubation with primary antibodies and horse radish peroxidase-conjugated anti-rabbit polyclonal antibody, sections were visualized with a DAB substrate (Boster, USA). Subsequently, the cells and tissue sections were observed using fluorescence microscopy.

### Colony formation assay

NAF cells or CAF cells were plated in a 5 cm dish at a cell density of 5 × 10^5^ cells/well. HCT116 colon cancer cells were co-cultured with a conditioned medium for 2 weeks until colonies were visible. After washing with PBS, cells were fixed with 4% paraformaldehyde and stained with 1% crystal violet. The stained colonies were scored using software.

### Tumor xenografts

Five-week-old male BALB/c nude mice were obtained from a Shanghai animal laboratory. All animal procedures were conducted in accordance with the guidelines approved by the Institutional Animal Care and Use Committee of Shanghai Jiao Tong University School of Medicine. Tumor xenografts were generated by subcutaneously co-injecting human fibroblasts with HCT116 cells. The ratio of fibroblasts to HCT116 cells was 3:1, and the number of cancer cells in each injection was 1 × 10^6^ cells. The injections were made bilaterally into the armpits.

Non-activated fibroblasts (NAF) and CAFs were treated with radiation before injection at 6 Gy. After 6 weeks, the mice were sacrificed, and the tumors were excised. The tumor volume was calculated using digital caliper measurements and the following formula: tumor volume (mm^3^) = ½ × longest diameter^2^ × shortest diameter.

### ATAC-sequencing, CUT-Tag, and RNA-sequencing analysis

Cells were fixed in 1% formaldehyde for 10 min and quenched by adding 2 M Glycine to a final concentration of 240 mM. Chromatin was immunoprecipitated from sonicated cell lysates using antibodies against H3K27Ac, H3K27me3, H3K4me3, RNA pol II, Med1, or KDM6A, as well as IgG, with the SimpleChIP® Enzymatic Chromatin IP Kit according to the manufacturer’s instructions.

For RNA-seq analysis, quality control was performed using fastqc v0.11.9, and the reads were aligned to the human reference genome (GRCh38 version) using HISAT2. Subsequently, Stringtie v2.2.1 software was used to obtain a gene counts matrix, and gene expression was evaluated using transcripts per million (TPM). The standard DEseq2 workflow was used to identify differentially expressed genes (DEGs). *P*-values were computed using the Wald test and adjusted (padj) for multiple testing using the Benjamini and Hochberg methods. Differentially expressed genes (DEGs) with |log2-fold change| > 1 and *P*-value < 0.05 were visualized in a heatmap based on log-transformed and scaled gene expression.

For data analysis of ATAC-seq and Cut&Tag, raw sequences were processed using fastqc v0.11.9 for quality control. Adapter sequences and low-quality sequences were removed using cutadapt v3.4. The clean reads were aligned to the human reference genome (GRCh38 version) using bowtie2 with default parameters. Uniquely and appropriately paired mapping reads were selected, while reads from mitochondrial DNA and duplicate sequences were excluded. The remaining reads were stored in BAM files for further analysis. The BAM files can be transformed into bigWig files using deepTools 3.5.1 to visualize the distribution of reads among the chromatin. Peak calling was performed using macs2 with default parameters, and overlapping or differentially enriched peaks between two conditions were determined using bedtools v2.

### AOM/DSS-induced colon cancer

The AOM/DSS-induced colon cancer model was established using 5-week-old male ICR mice. AOM (Sigma, USA) was administered via intraperitoneal injection at a dose of 10 mg/kg on the first day of the first week. After one week, mice were given 2.5% (w/v) DSS (YEASEN, China) dissolved in drinking water on the second, fifth, and eighth weeks. Approximately 70 days later, GSK-J4 and anti-IGF1 were injected into the mice. Verification of AOM/DSS-induced colon cancer was conducted using enteroscopy and 18F-FDG micro-PET/CT.

### Co-immunoprecipitation

Immunoprecipitation (IP) was performed using pol II, Med1, KDM6A antibodies, or nonspecific immunoglobulin G (IgG), following the manufacturer’s protocol from Cell Signaling Technology. The products were separated by SDS–polyacrylamide gel electrophoresis and further analyzed through silver staining (Sangon Biotech, China) and western blotting. Specific bands in the silver-stained gels were excised for protein identification using liquid chromatography-tandem mass spectrometry (MS) analysis.

### Statistical analysis

The data are presented as the medians ± SD. The unpaired two-tailed *t*-test and two-way ANOVA were used as indicated. Statistical significance was defined as *P* < 0.05 unless otherwise stated. Each experiment was repeated independently with similar results.

## Supplementary information

Supplemental figures

Original data

Supplemental Table 1

## Data Availability

All other data information may be obtained from the corresponding author upon reasonable request. The RNA and ATAC sequences have been deposited at NCBI under accession numbers PRJNA1077791 and PRJNA1077897, respectively. The accession numbers of the ChIP-seq dataset and the scRNA-seq dataset from the GEO depository are GSE283546 and HRA000979, respectively. The accession number of the proteomics data from the ProteomeXchange depository is PXD058594.
