# Supplementary material for: KDM6A downregulation promotes tumor-prone cytokines expression in cancer-associated fibroblasts by activating enhancers
Source: Cell Death Dis. 2025 Jul 14;16(1):523. doi: 10.1038/s41419-025-07818-3 (PMC12259948; doi:10.1038/s41419-025-07818-3)
Supplement: Supplementary file 1 — Supplemental figures [file 41419_2025_7818_MOESM1_ESM.pdf]

Figure S1

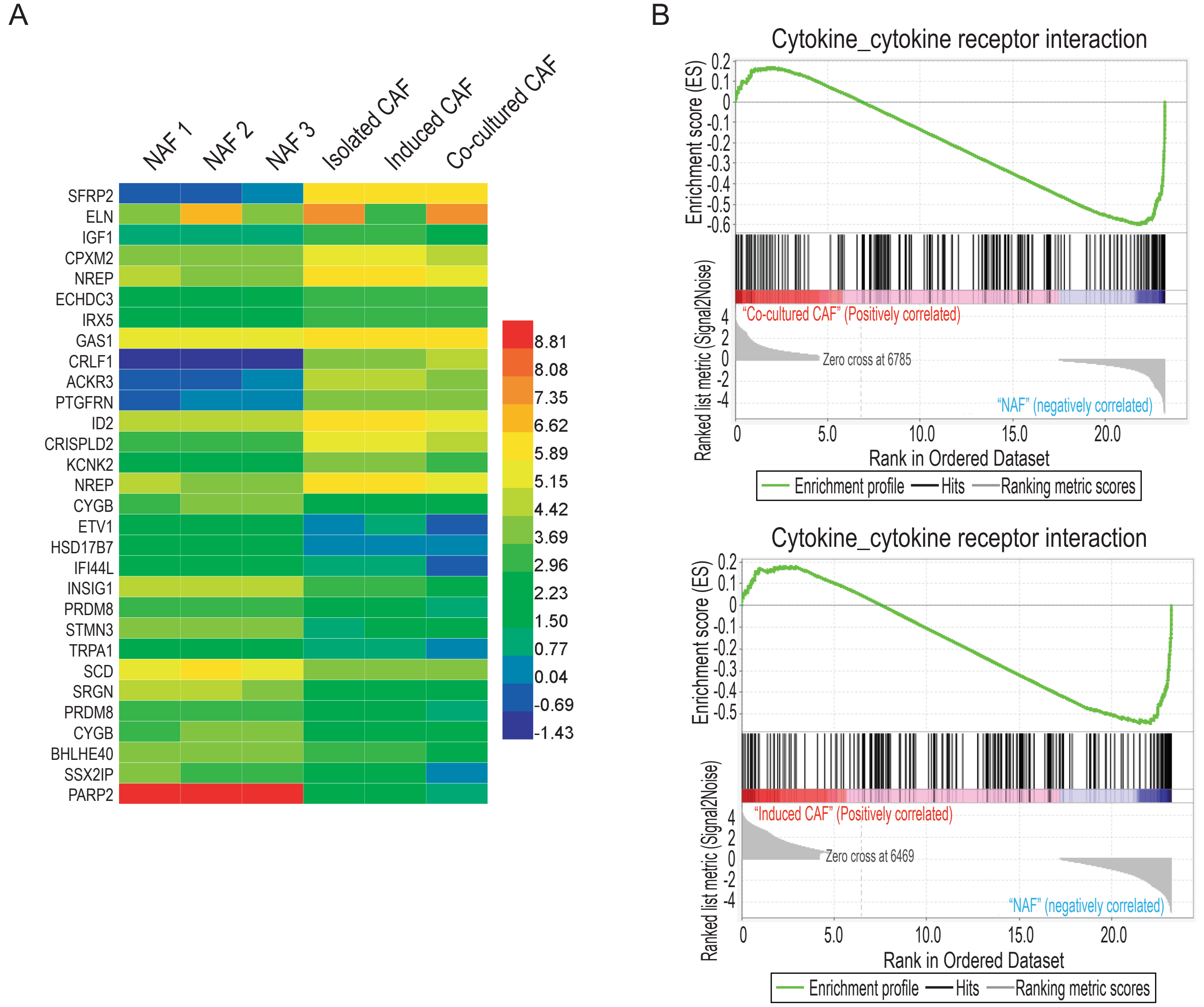

Figure S1 legend:

A. Heatmap of representative secreted cytokines in various NAFs and CAFs.

B. The GSEA analsis of genes involved in the cytokine_cytokine receptor interaction in PDGF-induced CAF and TGF-β-induced CAF.

Figure S2

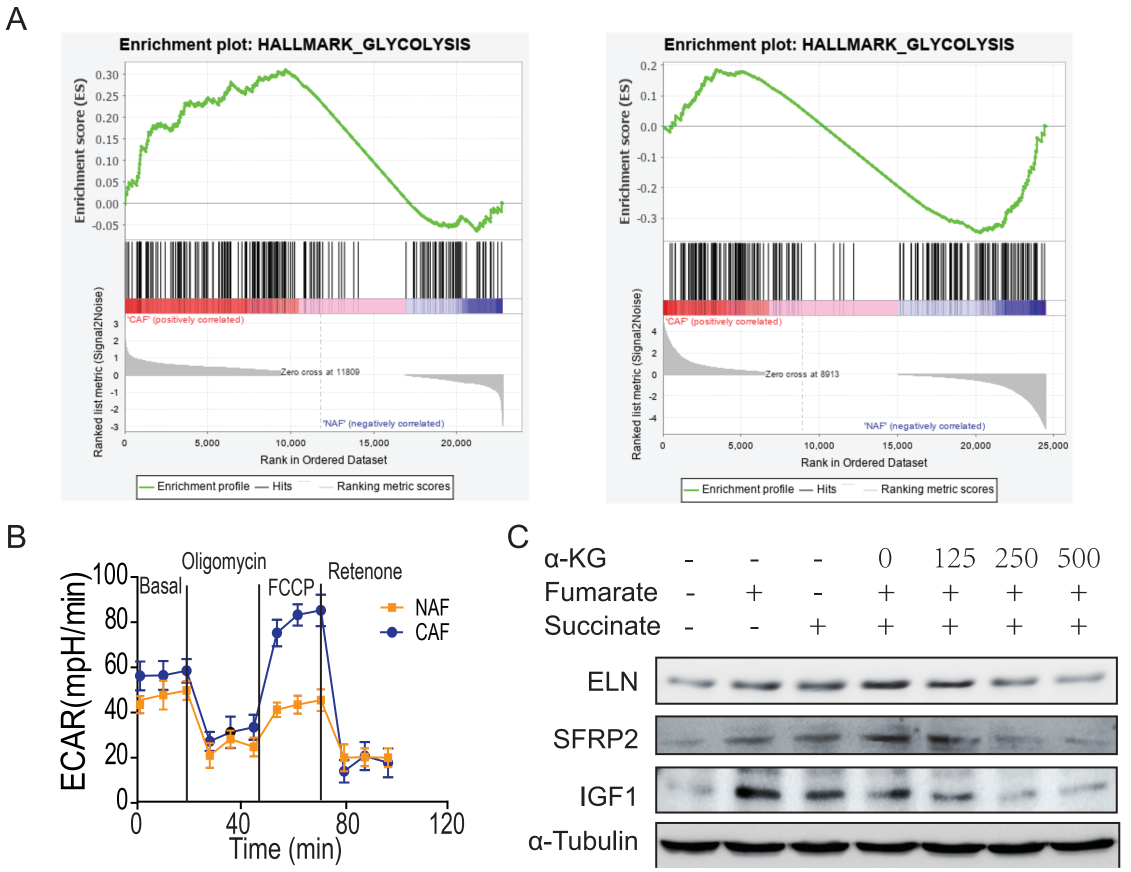

Figure S2 legend:

A. The GSEA analisis of genes involved in glycolysis pathway in PDGF-induced CAF and TGF-β-induced CAF.

B. Determination of extracellular acidificaion rate (ECAR) in CAFs and NAFs.

C. Imunoblotting detection of ELN, SFRP2, IGF1 expression in NAFs treated with fumarate, succinate or α-KG alone or combination. The concentration of fumarate and succinate was 40 μM and 1000 μM, respectively.

Figure S3

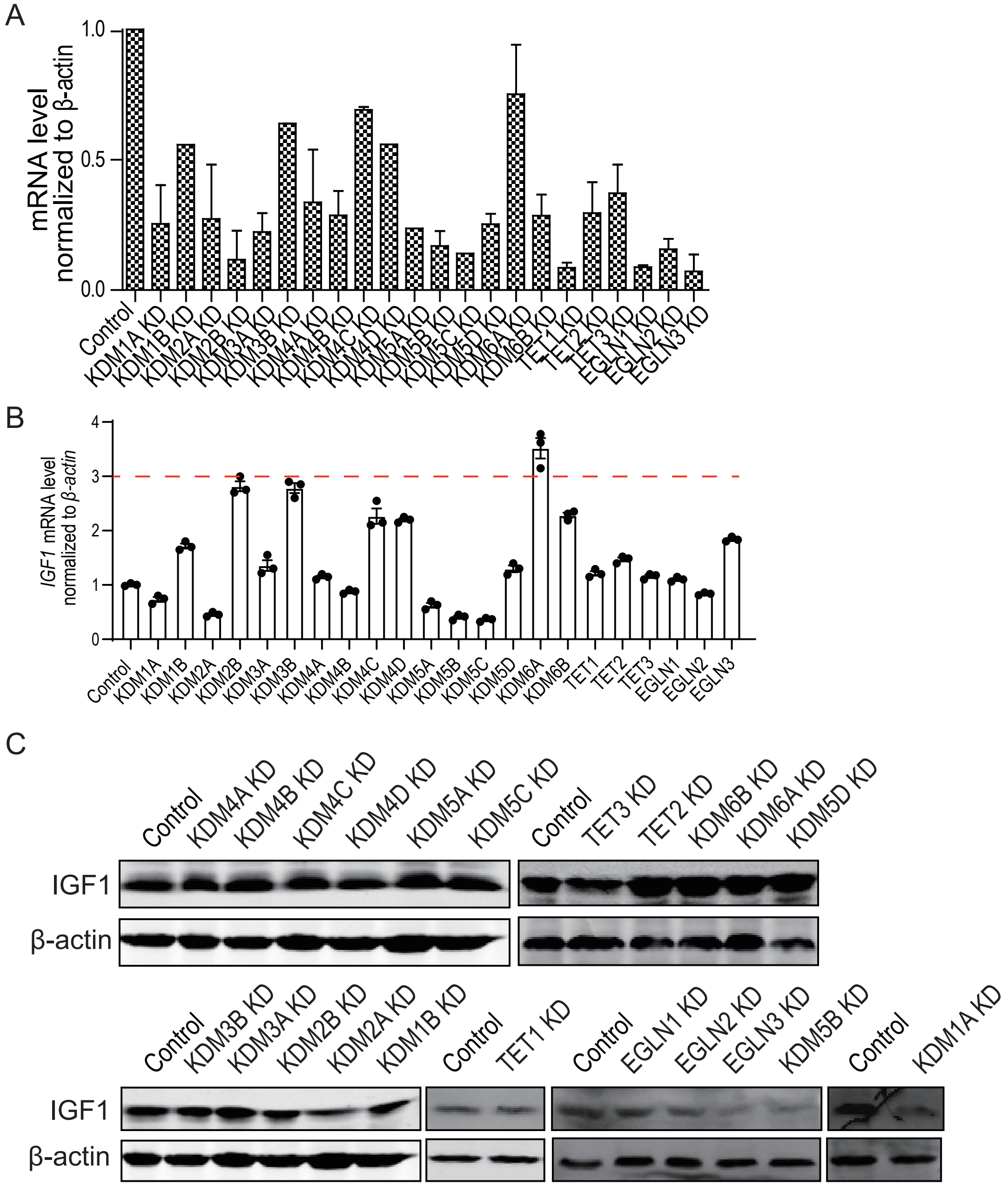

Figure Legend

A. Knockdown efficiency analysis for α-KG-dependent dioxygenases in NAF using a mixed shRNA pool.

B. Screening of α-ketoglutarate dependent dioxygenase(s) regulating IGF1 expression by mixed shRNA pools targeting these dioxygenases.

C. Immunoblotting analysis of IGF1 expression in NAFs depleted of various dioxygenases.

Figure S4

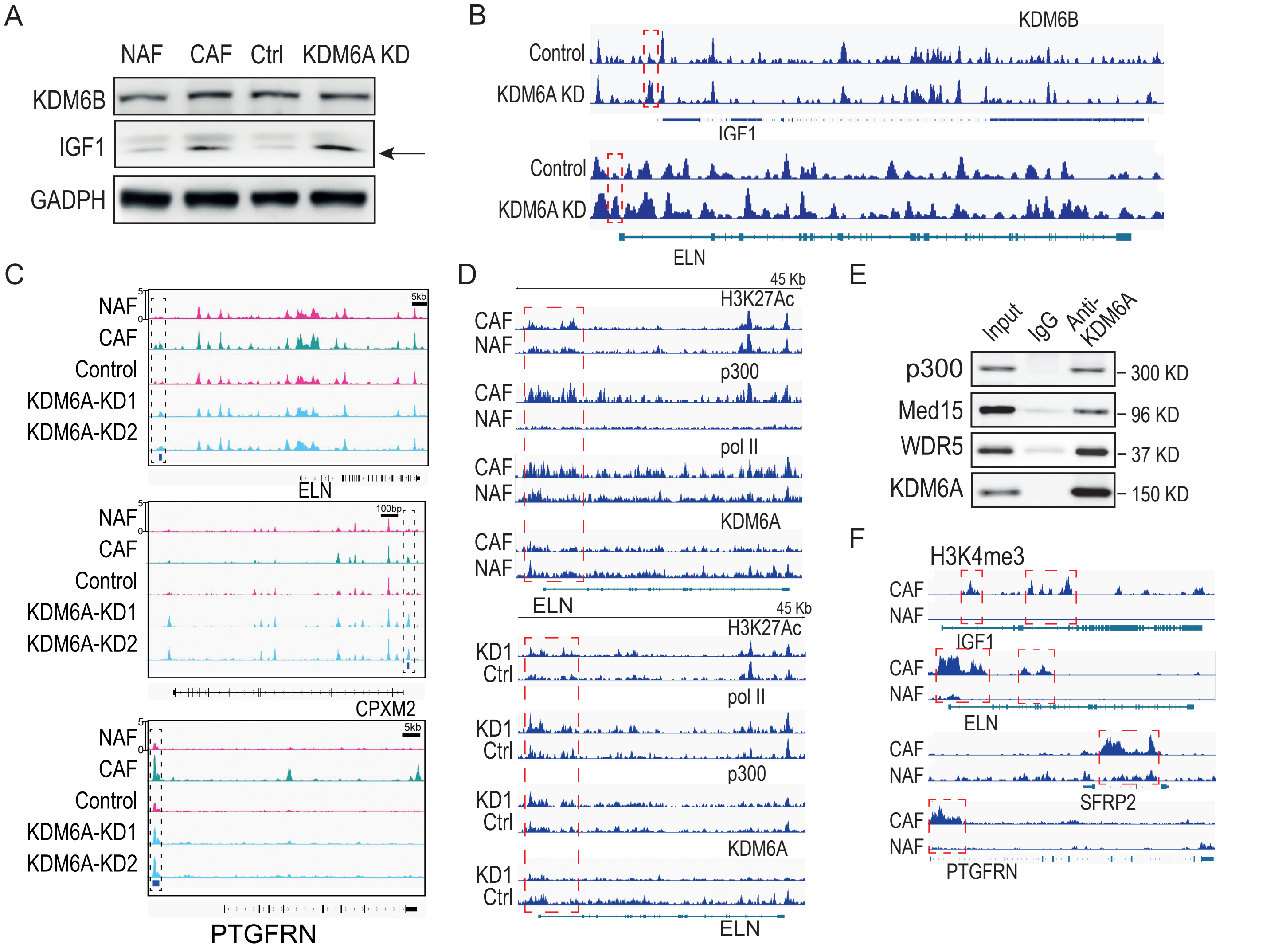

Figure S4 legend:

A. Immunoblotting was used to detect the expression of KDM6B in CAFs and KDM6A-depleted NAFs.

B. KDM6B enrichment on the promoters of IGF1 and ELN in CAFs with or without depletion of KDM6A.

C. The chromatin accessibility of ELN, CPXM2, and PTGFRM was detected by ATAC-sequencing in NAF, CAF, KDM6A-depleted NAFs.

D. Assessment of H3K27ac, p300 and pol II enrichment at the promoter region of ELN in the CAFs (upper panel) and KDM6A-knockdowned NAFs (lower panel) by CUT-Tag technology.

E. Evaluation of KDM6A association with p300, Med15 and WDR5 by co-immunoprecipitation.

F. Assessment of H3K4me3 enrichment at the promoter regions of IGF1, ELN genes by CUT-Tag.

Figure S5

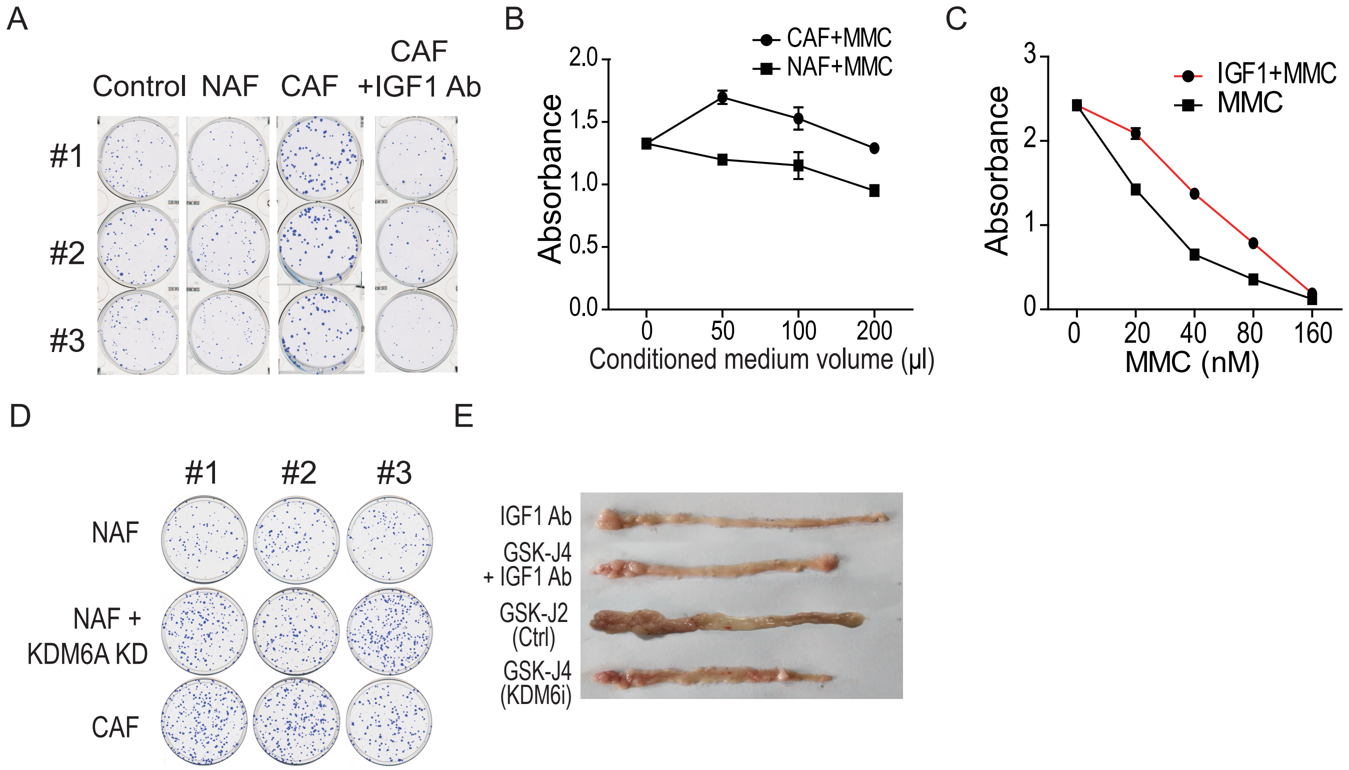

Figure S5 legend

A. Evaluation of the colony formation of colorectal cancer cells. HCT116 cells were co-cultured with fibroblast-conditioned media for 10 days. The content of IGF1 antibody was 1 μg/ml.

B. Determination of the impact of CAF on HCT116 cell resistance to mitomycin C. The dose of MMC was 50 nM.

C. Determination of the influence of IGF1 on HCT116 cell resistance to mitomycin C. The concentration of IGF1 was 20 ng/ml.

D. Assessment of the effect of conditioned media on the colony formation of coloreactal cancer cells. HCT116 cells were co-cultured with conditioned media of KDM6A-depleted NAF or CAF for 10 days.

E. Representative images of the colon burden tumors in mice after the designated treatment.
