# Supplementary figures and images for: KDM6A downregulation promotes tumor-prone cytokines expression in cancer-associated fibroblasts by activating enhancers
Source: Cell Death Dis. 2025 Jul 14;16(1):523. doi: 10.1038/s41419-025-07818-3 (PMC12259948; doi:10.1038/s41419-025-07818-3)

Figure 1

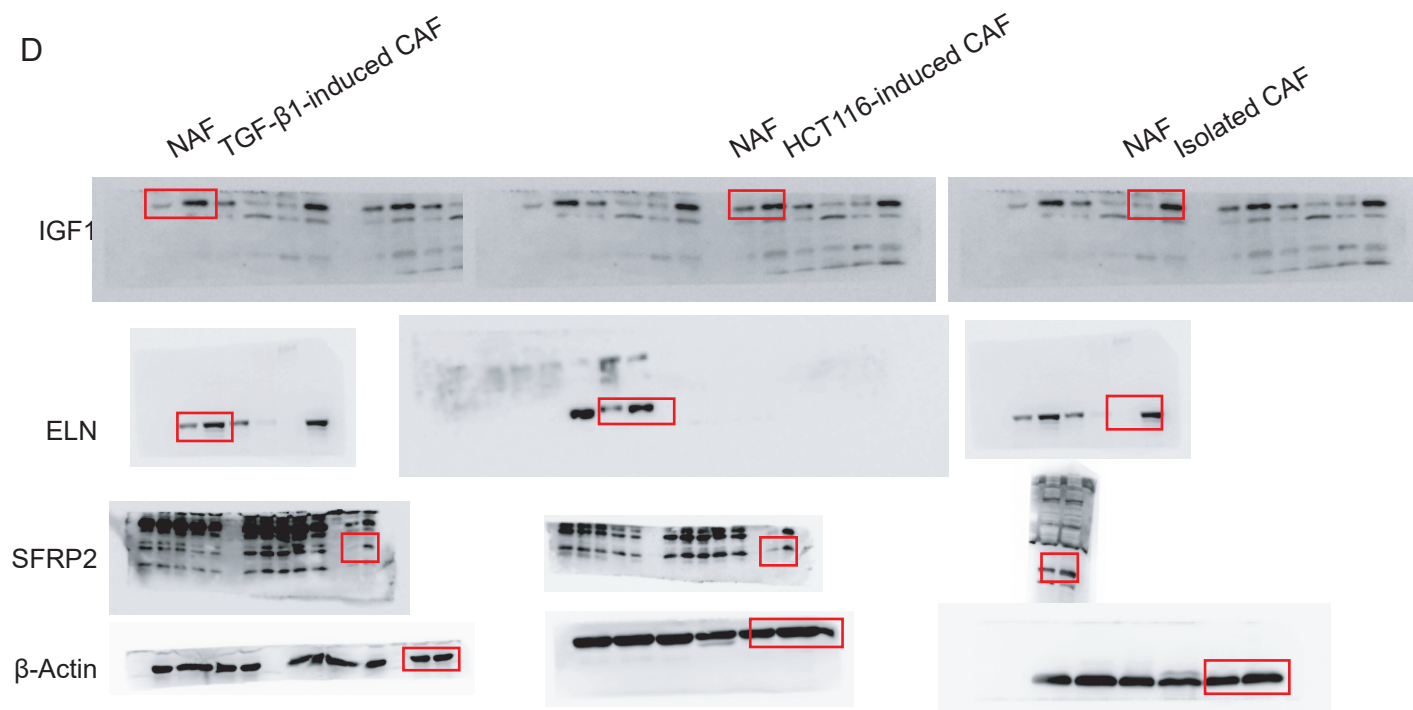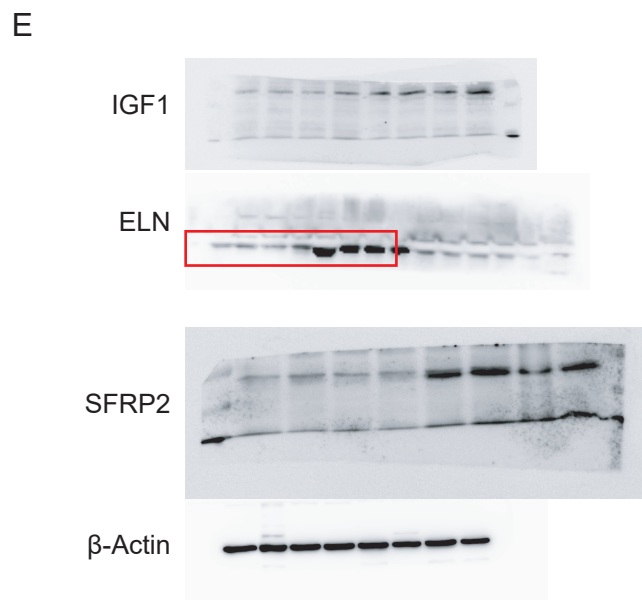

Figure 2

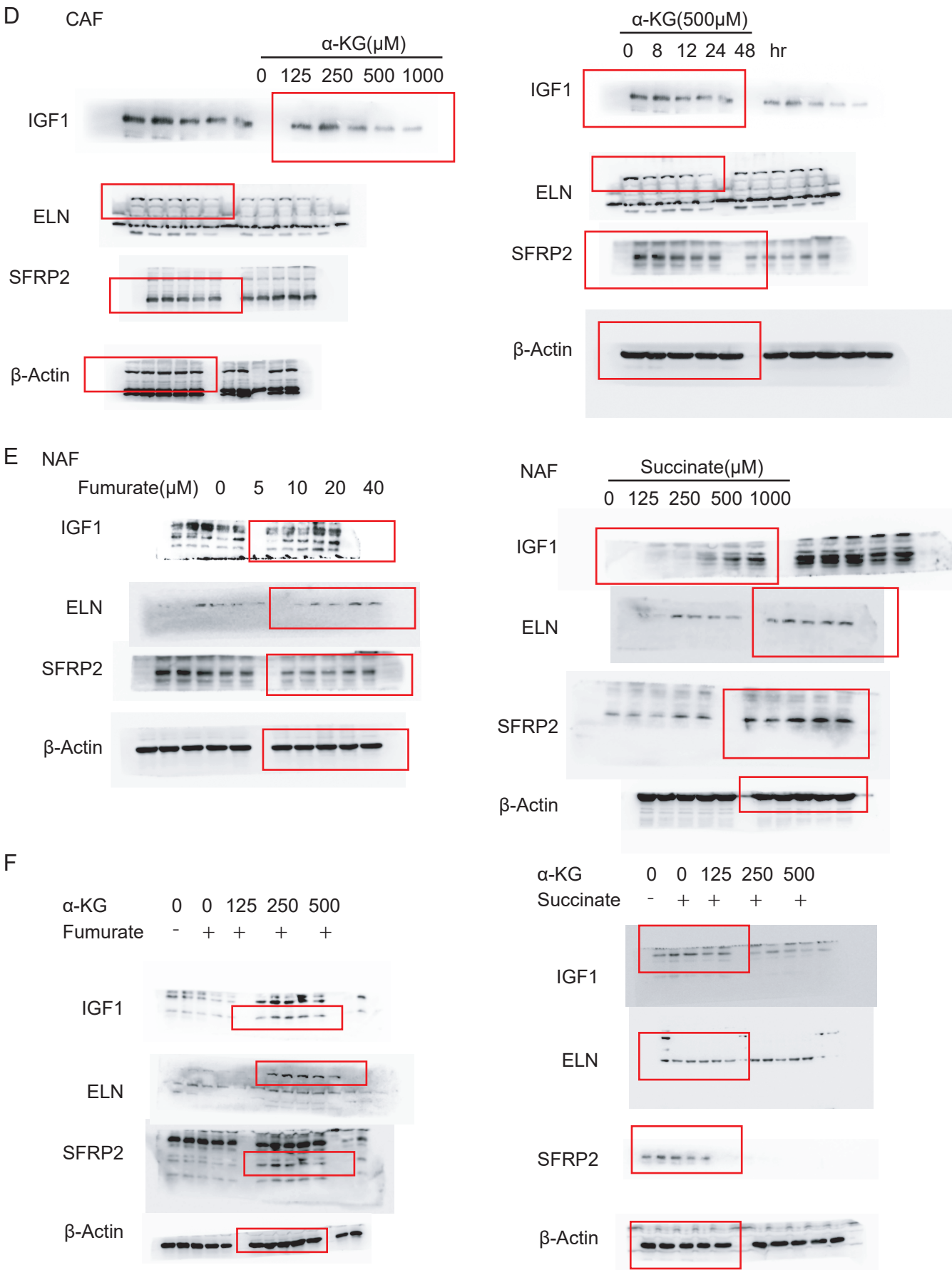

Figure 3

C

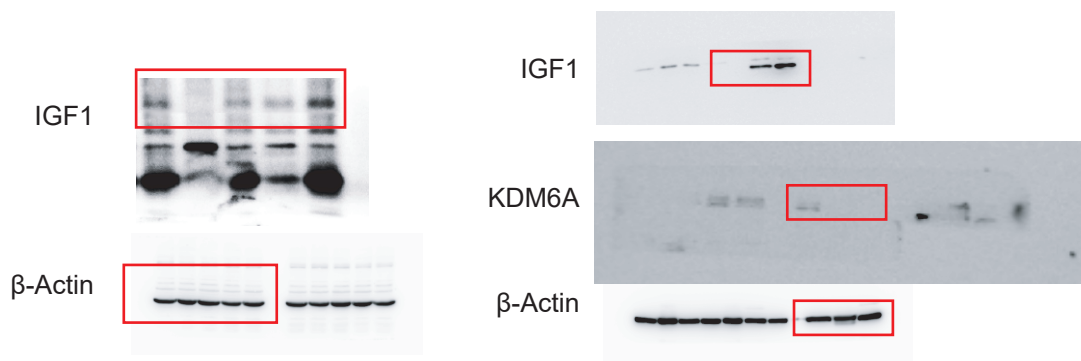

D

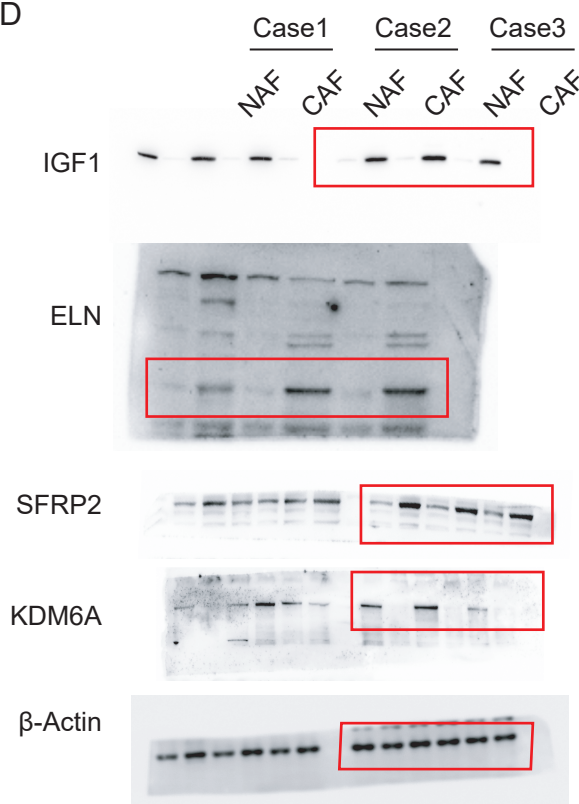

F

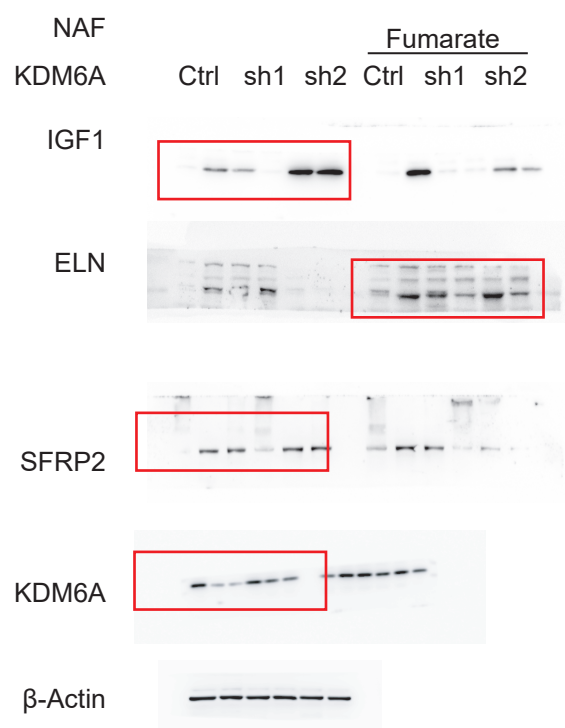

Figure 5

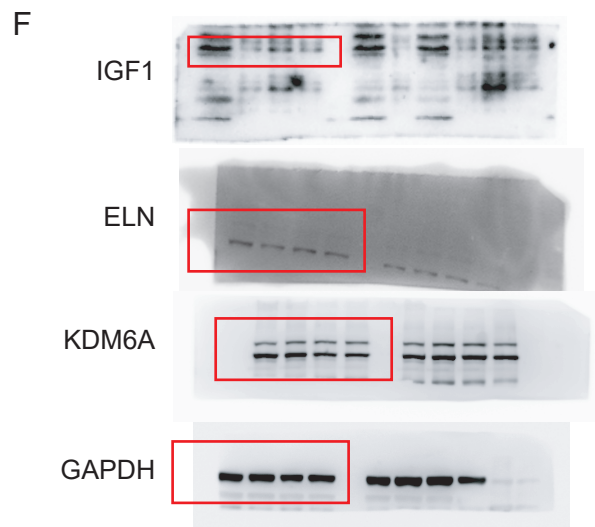

Supplement: Supplementary file 2 — Original data [file 41419_2025_7818_MOESM2_ESM.pdf]
