# Supplementary material for: KDM6A downregulation promotes tumor-prone cytokines expression in cancer-associated fibroblasts by activating enhancers
Source: Cell Death Dis. 2025 Jul 14;16(1):523. doi: 10.1038/s41419-025-07818-3 (PMC12259948; doi:10.1038/s41419-025-07818-3)
Supplement: Supplementary file 3 — Supplemental Table 1 [file 41419_2025_7818_MOESM3_ESM.docx]

| Antibody | Catalogue Number | Company |
| --- | --- | --- |
| anti-FAP | ab207178 | Abcam |
| anti-FSP1 | 13018 | Cell Signaling Technology |
| anti-IGF1 | A11985 | Abclonal |
| Anti-ELN | A2723 | Abclonal |
| Anti-KRT20 | A19041 | Abclonal |
| Anti-SFRP2 | A5383 | Abclonal |
| Anti-KDM6A | 33510S | Cell Signaling Technology |
| Anti-KDM6B | A01309 | Boster |
| anti-Med1 | A300-793A | Bethyl Laboratories |
| anti-H3K27me1 | 61016 | active motif |
| anti-H3K27me3 | 39155 | active motif |
| anti-H3K27ac | 39133 | active motif |
| anti-H3K4me3 | 39060 | active motif |
| Anti-p300 | ab14984 | Abcam |
| anti-Med15 | 11566-1-AP | proteintech |
| anti-WDR5 | ab56919 | Abcam |
| Anti-Pol II | Sc-56767 | SantaCruz |
| anti-β-actin | AC048 | Abclonal |
| Anti-α-Tubulin | A6830 | Abclonal |
| HRP-conjugated Goat anti-Rabbit IgG | AS014 | ABclonal |
| HRP-conjugated Goat anti-Mouse IgG | AS003 | ABclonal |

Table 1: Antibody list
